# *Ribes himalense* as potential source of natural bioactive compounds: Nutritional, phytochemical, and antioxidant properties

**DOI:** 10.1002/fsn3.2256

**Published:** 2021-05-03

**Authors:** Qing Sun, Na Wang, Wenhua Xu, Huakun Zhou

**Affiliations:** ^1^ Northwest Institute of Plateau Biology Chinese Academy of Sciences Xining China; ^2^ University of Chinese Academy of Sciences Beijing China; ^3^ Key laboratory of Restoration Ecology of Cold Area in Qinghai Province Xining China

**Keywords:** antioxidant activity, chemical composition, nutritional value, *Ribes himalense*, small berry

## Abstract

*Ribes himalense* Royle ex Decne. (family *Saxifraaceae*, subfamily *Grossulariaceae*, genus *Ribes*) is a wild berry fruit with illustrated health‐promoting features, which widely distributed in Northwest China are deficiently exploited. This study aimed to assess the potential of a *Ribes himalense* as a source of natural bioactive compounds through characterizing its nutraceutical characteristics, phytochemicals properties, and antioxidant ability. Fresh berries were quantitatively analyzed for proximate composition, minerals, vitamins, amino acids, total polyphenols, total flavonoids, anthocyanins, procyanidin, and polysaccharides contents through China National Food Safety Standard; the characterization and identification of extracts of wild berries obtained with ethanol 30%, ethanol 50%, and ethanol 95% were firstly performed by UPLC‐Triple‐TOF‐MS^2^. Furthermore, antioxidant activity of the ethanol extract was evaluated via different assay methods such as DPPH, ABTS, and FRAP. The results indicated that the most important bioactive composition was procyanidin (0.72%), polyphenols (0.49%), total flavonoids (0.38%), vitamin C (64.6 mg/100g FW), and K (218.44 mg/100 g FW), and a total of 95 compounds were detected with polyphenols, flavonoids, and proanthocyanidins as the dominant, and also ethanol extract possessed stronger antioxidant activity. These results suggested that *Ribes himalense* fruit has great potential in protecting human health, with the focus on the development of functional products.

## Graphical abstract

(1) Unambiguously recognized by comparing with reference standard compounds, such as analysis of nutrients content (including minerals, amino acids, vitamins); (2) characterized by cleavage pathways and typical fragment ions according to the relevant references; (3) preliminarily identified by searching Scifinder and Reaxy databases, like phenolic acids, flavonoids, proanthocyanidins, anthocyanins, and organic acids. These results suggested that this fruit has great potential in protecting human health, with the focus on the development of functional products.

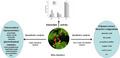

## 1 INTRODUCTION

Berries reflect the most widely eaten fruits in the human diet, such as blueberry (*Vaccinium corymbosum*), strawberry (*Fragaria ananassa*), blackberry (*Rubus fruticosus*), raspberry (*Rubus idaeus*), cranberry (*Vaccinium macrocarpon*), sea buckthorn (*Hippophae rhamnoides*), goji (*Lycium barbarum*), black currant (*Ribes nigrum*), red currant (*Ribes rubrum*), white currant (*Ribes pallidum*), and white and red gooseberries (*Ribes grossularia*) are considered a good source of nutrients (vitamins, amino acids, and minerals) and bioactive compounds (polyphenols, pigments, anthocyanins, flavonoids, sugars, lignans, and fatty acids) (Neri‐Numa et al., 2018; Olas, 2017; Zorzi et al., 2020). On one hand, there is growing evidence that edible small berry fruits may have great potential for multiple health benefits (Schreckinger et al., 2010; Seeram, 2008, 2011; Stoner et al., 2007). Because small berries have antioxidant, anti‐inflammatory, and antibacterial activities (Manganaris et al., 2013), their ingredients and health‐promoting properties are related to the prevention or delay of age‐related chronic diseases, so they are rapidly becoming popular in Western countries (Balogh et al., 2010). On the other hand, berries are rich in nutrients, such as fibers, amino acids, minerals, and vitamins, the large number of bioactive ingredients (including phenols and flavonoids) present in these fruits, which are believed to have various health benefits (Baliga & Dsouza, 2011; Battino et al., 2009; Bishayee et al., 2011; Seeram, 2010; Zafra‐Stone et al., 2007). In addition, clinical evidence regarding the potential health benefits of berries shows that improve the postprandial blood glucose, ameliorate distribution of inflammation markers, and enhance antioxidative ability of plasma by acute consumption; long‐time intake may ameliorate plasma lipid status, decrease chronic inflammation, and sustain cardiovascular health (Yang & Kortesniemi, 2015). Most of the researches have focused merely on “mainstream” berries such as blueberries and strawberries, and future studies should be expanded to small berries with initial health‐promoting potential, such as sea buckthorn, black currant, and *Ribes himalense*.

*Ribes himalense* Royle ex Decne. plant is a perennial deciduous shrub in *Saxifraaceae* family (Sun & Xu, 2019). It is widely distributed in northwest areas of China, such as Qinghai, Shaanxi, Tibet, and Gansu. The fruit is a small edible berry, usually used as a common Chinese herbal medicine “Saiguo” in Tibet, which used to treat various vascular diseases and hepatitis for a long time [in Chinese] (Xiang et al., 2021). Some *Ribes* species fruits (such as black currant and *Ribes uva‐crispa*) are often used to make jams, juices, preserves, fermented beverages, and wine because of their rich nutritional properties (Kendir et al., 2019; Orsavová et al., 2019). Furthermore, genus *Ribes* has been extensively studied mainly due to a variety of bioactive compounds with potential health benefits, such as flavonoids, organic acids, polysaccharides, and phenolic acids (Lyashenko et al., 2019; Shaw et al., 2017; Tian et al., 2017). However, *R*. *himalense* fruit is an underutilized fruit that is rarely obtained because it is located in cold regions at high altitudes. The lack of scientific evidence on its nutritional value and health benefits has also hindered the promotion and application of this wild fruit in modern foods and medicines. In addition, the high yield of wild *R. himalense* fruit makes it an inexhaustible renewable resource source. Furthermore, few studies have been conducted on the nutrient, chemical composition, and antioxidant activity of the *R*. *himalense* fruit. In view of this, the purpose of this study is to reveal the characteristics of nutritional value, phytochemical compositions, and antioxidant in *R. himalense* fruit. Various profiles, such as nutrients (moisture, protein, fat, crude fiber, elements, vitamins, and amino acids) and bioactive compounds (polyphenols, flavonoids, proanthocyanidin, polysaccharide, anthocyanidin), were further investigated on the exploitation of *R. himalense* resource. Assessing the chemical composition of different ethanol concentrates from *R. himalense* berries in China through high‐resolution liquid mass spectrometry (UPLC‐Triple‐TOF‐MS^2^). Meanwhile, the antioxidant properties in vitro of three different ethanol extracts were comprehensively evaluated via three common antioxidant capacity methods (DPPH, ABTS, and FRAP). The findings obtained will be useful for the future development of functional products using the fruit in the nutritional food, pharmaceutical, and cosmetic industries.

## 2 MATERIALS AND METHODS

### 2.1 Plant materials

Fresh Fruits of *R*. *himalense* were collected from the Marque river forest farm, Banma County, Qinghai, China. The fruits were authenticated by an associate professor Wenhua Xu in Northwest Institute of Plateau Biology, Chinese Academy of Science, Qinghai province, China. The berries were rinsed completely with ultrapure water. The juice was oppressed from the fruits using a juicer, and the juice concentrates extracted were stored at 4°C in sealed containers until next use.

### 2.2 Chemicals and reagents

The quality of all chemicals was of analytical grade or stated otherwise. All the chromatographic solvents were GC grade for GC analysis. Water was obtained from a Milli‐Q purification system (Millipore, North Ryde, NSW, Australia).

### 2.3 Proximate analysis

The moisture content (GB 5009.3‐2016), the protein content (GB 5009.5‐2016), the fat content (GB 5009.6‐2016), and the crude fiber content (GB/T 5009.10‐2003) were determined according to China National Food Safety Standard.

### 2.4 Amino acid analysis

After adding 6 ml of 1:1 hydrochloric acid to the 0.05 g sample, continue to add 3–4 drops of phenol, freeze for 3–5 min, repeat vacuum‐filling with nitrogen 3 times, and hydrolyze for 22 hr. Open the hydrolysis tube, filter the acid hydrolyzate into a 50‐ml beaker, evaporate it in a water bath, and dilute to 2.0 ml with 20 mmol/L hydrochloric acid. Take 0.5 ml of the hydrolyzed solution into a 10‐ml volumetric flask, add 1 ml of 0.5 M Na_2_CO_3_ solution, and add 1 ml of 0.1% 2,4‐dinitrofluorobenzene 60°C water bath to derive for 1 hr, and use 0.1 M KH_2_PO_4_ to make the volume to 10 ml. To be tested after filtration with 0.45 μm inorganic membrane. The amino acid analysis was performed on a Waters 1525 Series HPLC system (Waters Corp., USA) equipped with a binary gradient pump and a VWD detector. The separation was carried out at 37°C using a Phenomenex Gemini‐NX column (250 × 4.6 mm, 5 μm). Mobile phase A was acetonitrile/H_2_O (50:50, v/v), and mobile phase B was 0.05 mol/L sodium acetate solution (6.80 g sodium acetate trihydrate was dissolved in 900 ml water, adjust the pH to 4.0–5.0 with glacial acetic acid, and dilute to 1,000 ml with water), gradient elution for 45 min. The flow rate of 1 ml/min, the injection volume was 10 μl, and the detection wavelength was 360 nm.

### 2.5 Mineral content determination

2.5 g samples were weighed into PTFE inner tank and added with 10 ml nitric acid, cover the safety valve and soak overnight, and digested in microwave digestion instrument. When the digestive liquid was colorless and transparent or slightly yellow, cooled, constant volume with ultrapure water to 25 ml, mix well for later use. The minerals were determined according to the standard method of China National Food Safety Standard (GB 5009.268‐2017). Potassium (K), calcium (Ca), magnesium (Mg), iron (Fe), zinc (Zn), manganese (Mn), and copper (Cu) contents were determined by using an inductively coupled plasma spectrometer (ICPS, OES‐725, USA).

### 2.6 Vitamins determination

#### 2.6.1 Vitamin B1

The vitamin B1 (VB1) was analyzed using China National Food Safety Standard method (GB 5009.84‐2016). VB1 was extracted with 60 ml 0.1 mol/L hydrochloric acid and 2.0 ml mixed enzyme solution (1.76 g papain and 1.27 g amylase, with water constant volume to 50 ml). VB1 was determined by a high‐performance liquid chromatography (HPLC) method. A Waters 1525 Series HPLC system equipped with a fluorescence detector and a Dikma Spursil C_18_‐EP column (250 × 4.6 mm, 5 μm). Mobile phase was 0.05 mol/L sodium acetate solution and methanol (65:35, v/v) with gradient elution for 12 min, excitation wavelength 375 nm, emission wavelength 435 nm, flow rate of 0.8 ml/min, and injection volume of 10 μl.

#### 2.6.2 Vitamin B2

The vitamin B2 (VB2) was determined by a HPLC method (GB 5009.85‐2016). Vitamin B_2_ was extracted using 60 ml 0.1 mol/L hydrochloric acid and 2 ml mixed enzyme solution (2.345 g papain and 1.175 g taka‐diastase, with water constant volume to 50 ml) and was determined by a Waters 1525 Series HPLC system equipped with a fluorescence detector and a Dikma Spursil C_18_‐EP column (250 × 4.6 mm, 5 μm). Mobile phase was 0.05 mol/L sodium acetate solution and methanol (65:35, v/v) with gradient elution for 14 min, excitation wavelength 462 nm, emission wavelength 522 nm, flow rate of 1 ml/min, and injection volume of 10 μl.

#### 2.6.3 Vitamin C

The vitamin C (ascorbic acid) was analyzed by a HPLC method (GB 5009.86‐2016), which a Shimadzu LC‐2030C 3D Series HPLC system equipped with a PDA detector and a Shim‐pack GIST C_18_ column (250 × 4.6 mm, 5 μm). After the sample was dissolved in 20 g/L metaphosphoric acid, the absorbance was measured at 245 nm. Mobile phase was 20 mM metaphosphoric acid solution and methanol (98:2, v/v), gradient elution for 20 min, flow rate of 0.7 ml/min, and injection volume of 10 μl. The content of vitamin C was calculated on the basis of the calibration curve of L (+) ascorbic acid that acted as the standard reference.

#### 2.6.4 Vitamin E

The vitamin E (tocopherol) was extracted with 50 ml mixture of petroleum ether and ethyl ether (1:1, v/v) by shock for 5 min and extracted twice consecutively. The ether layer was combined. Wash the ether layer with 100 ml ultrapure water until the ether layer is washed to neutral, removing the lower aqueous phase. Filtrate the cleaned ether layer into 250 ml rotary evaporator through anhydrous sodium sulfate (about 3 g) and distill under reduced pressure in 40°C water bath. Remove the evaporator when there is about 2 ml ether solution left in the bottle and immediately blow dry with nitrogen. The residues in the evaporating flask were dissolved in methanol by stages and transferred to a 10‐ml volumetric flask. The solution was set to scale and passed through an organic mesofiltration membrane 0.22 μm for HPLC determination (GB 5009.82‐2016), which Agilent 1260 Series HPLC system equipped with a UV detector and a Dikma Spursil C_18_ column (250 × 4.6 mm, 5 μm). Mobile phase was methanol/H2O (98:2, v/v), gradient elution for 40 min, flow rate of 1 ml/min, and injection volume of 10 μl. The measurements at 294 nm against a blank sample, standard curves made with pure tocopherol were used for this purpose.

### 2.7 **Bioactive compounds analysi**s

#### 2.7.1 Determination of total phenolic content

The total polyphenol contents were determined according to spectrophotometric method of China Food Safety Standard (T/AHFIA005‐2018) which involved the reduction in Folin–Ciocalteu reagent by phenolic compounds. 1 ml of extract sample and standard (3,4,5‐trihydroxybenzoic acid) was incubated with 2.5 ml Folin–Ciocalteu's reagent, shaken, and then 2.5 ml of 15% (w/v) Na_2_CO_3_ was added and the solution hatched at 40°C water bath for 60 min, respectively. Absorbances of samples were measured at 778 nm using Cari‐300 Ultraviolet‐visible spectrophotometer (Varian Corp., USA). Gallic acid was used as the standard and formulated into standard series with concentrations of 0, 4, 8, 12, 20, and 30 mg/L, draw a standard curve, and the content of total polyphenols in the solution to be measured was calculated according to the standard curve. The results were indicated as gallic acid equivalent mg GAE/g fresh weight.

#### 2.7.2 Determination of total flavonoids content

The total flavonoids contents were investigated according to spectrophotometric method. Add 25 ml of 75% ethanol to 1 g of sample, sonicate for 30 min, filter into a 50‐ml volumetric flask, continue to add 75% ethanol to dilute to the mark, shake well, draw 2 ml of filtrate into a 50‐ml volumetric flask, and process according to the above steps, to get the sample solution which measures the absorbance at 510 nm using Cari‐300 Ultraviolet‐visible spectrophotometer, prepare a standard curve with rutin (0.2094 mg/ml) as a standard, and calculate the total flavonoid content. The total flavonoid content values were represented as rutin equivalents (RTE), that is, mg RTE/g fresh weight, and determined via a calibration curve.

#### 2.7.3 Determination of polysaccharide content

Weigh 0.5 g of the sample into a 150‐ml conical flask, add 50 ml of 75% ethanol and ultrasonic for 30 min, filter, add about 100 ml of water to the filter residue, heat, and boil on a heating plate for 30 min. Cool to room temperature, transfer the contents to a 250‐ml volumetric flask, add distilled water to wash the conical flask 3 times and transfer them into the volumetric flask together, add water to dilute to the mark, and shake well. Precisely pipet 3 ml, add 3 ml of Sevag reagent (chloroform: *n*‐butanol, 4:3, v/v), centrifuge at 9,000 rpm for 5 min, discard the middle denatured protein layer and the lower organic layer, and the aqueous phase continued to repeat the above operation until no denatured protein appears between the water phase and the organic layer. Dilute the solution 10 times, accurately pipette 0.5 ml of the solution into a test tube with stopper, add distilled water to make up to 2 ml, add 1 ml of 5% phenol solution blending, slowly add 5 ml of concentrated sulfuric acid, color reactions can be carried out after the shake, draw the standard curve with *D*‐anhydrous glucose as the standard, and use the ultraviolet spectrophotometer to determine the polysaccharide content of the sample at 490 nm.

#### 2.7.4 Determination of anthocyanin content

The content of anthocyanins was determined by Varian Cari‐300 Bio spectrophotometry using the pH differential method as described (Llivisaca et al., 2018). Put 75–100 mg of the sample in a 50‐ml brown volumetric flask, 25 ml extract (concentrated HCl: methanol, 4:96, v/v) was added, sealed, ultrasonic at 40°C for 30 min, and cooled to room temperature to obtain the test solution. According to the test solution: buffer solution (1:5, v/v), prepare two dilutions of the test solution, one of which is diluted with potassium chloride buffer (0.025 M, pH 1.0), and the other is used sodium acetate buffer (0.4 M, pH 4.5). Using distilled water as a blank control, equilibrate the two diluted solutions prepared above for 20 min, then measure the absorbance was then measured at λ_max_ and 700 nm in each solution, and the anthocyanin content was estimated using the following formula (Jiang, Yang, & Shi, 2017):

$$\text{Anthocyanins}\;\text{content}\;\left(\text{mg}/g\right)=\frac{A\times MW\times DF\times 10^{3}}{\varepsilon \times l}$$

where *A* = (*A*_λmax_ − *A*_700 nm_) pH 1.0−(*A*_λmax_ − *A*_700 nm_) pH 4.5; *MW* (Molecular Weight) = molecular weight of cyanidin 3‐glucoside is 449.2 g/mol; *DF* = dilution factor; *ɛ* = molar extinction coefficient of cyanidin 3‐glucoside is 29,600 L/mol·cm; *l* = path length, cm. All samples were analyzed in triplicate.

#### 2.7.5 Determination of proanthocyanidins content

Procyanidin and vanillin were purchased from Shanghai Chunyou Biotechnology Co. (Shanghai, China). The content of procyanidin in the extract solution was determined using the standard vanillin‐Hydrochloric acid method (Fang et al., 2020). Vanillin/methanol solution (4 g/100 ml) of 3 ml and concentrated HCl of 1.5 ml were added to 1 ml of the extract solution in a 25 ml tube with plug (out of light), mixed well. Color development at room temperature for 15 min, with methanol as the control. The absorbance was measured at 500 nm using a Cari‐300 Bio Ultraviolet‐visible spectrophotometer, and the content of procyanidins was calculated by standard curve method.

### 2.8 UPLC‐Triple‐TOF/MS analysis

#### 2.8.1 Extract preparation

Fresh fruit was pressed to produce fruit juice, which was eluted with 30% ethanol with AB‐8 macroporous resin to obtain component 1 (Fr 1). Component 2 (Fr 2) was obtained by 60% ethanol elution. Component 3 (Fr 3) was obtained by eluting with 95% ethanol. The extracts were stored at 4°C prior to further assay. Three components were concentrated by rotary evaporation, and 4–5 ml of which were absorbed, respectively, into the EP tube to obtain three samples. The samples were treated as follows: Fr1 samples were concentrated to dry, followed by 10 ml 50% acetonitrile‐aqueous solution, ultrasonic for 2 min, centrifugation at 10,000 rpm for 20 min, and the supernatant was taken for testing; Fr2 samples were ultrasonic for 5min, centrifuged at 10,000 rpm for 30 min, and the supernatant was taken for testing; the Fr3 sample was concentrated to dry, then 2ml 50% acetonitrile‐aqueous solution was added, followed by ultrasonic for 2 min, centrifugation at 10,000 rpm for 20 min, and the supernatant was taken for testing.

#### 2.8.2 Equipment

UPLC‐Triple‐TOF/MS system: Acquity™ ultra high‐performance liquid chromatograph (Waters Corp., USA), Triple TOF 5600^+^ time‐of‐flight mass spectrometer, equipped with electrospray ion source (AB SCIEX Corp., USA); Eppendorf minispan centrifuge (Eppendorf Corp., Germany).

#### 2.8.3 Test Conditions

Fr1 chromatographic conditions: Waters ACQUITY UPLC HSS T3 column (150 mm × 3.0 mm i.d., 1.7 µm) was used; 0.1% formic acid solution was used as mobile phase A, 0.1% formic acid added acetonitrile was used as mobile phase B. The elution was 5% of mobile phase B at the beginning and increased linearly to 40% B at 32 min and increased to 95% B at 36 min; the re‐equilibration postrun time was 10 min. Fr1 was monitored by UV detection at 254 nm for compounds. The column temperature was 50°C, and an injection volume was 2 μl with a flow rate of 0.3 ml/min.

Fr2 and Fr3 chromatographic conditions: The samples were subjected to an Acquity™ UPLC system equipped with a tunable UV (TUV) detector, and an ACQUITY UPLC HSS T3 column (150 mm × 3.0 mm i.d., 1.7 µm), and at a column temperature of 50°C. The solvent system consisted of 0.1% formic acid (A) and 0.1% formic acid acetonitrile (B) with the following linear gradient elution: 5%–40% B (from 0 to 22 min), 40%–95% B (from 22 to 33 min), and keeping 95% B (from 33 to 36 min). The compounds were detected at 254 nm, at a flow rate of 0.3 ml/min, and the injection volume was 5 μl.

Fr1, Fr2, and Fr3 mass spectrometry conditions: The Triple TOF 5600^+^ MS was conducted in a negative ionization scanning mode with the optimized parameters as follows: the mass scanning range was m/z 100–1,500; atomizing gas (GS_1_): 55 psi; atomization gas (GS_2_): 55 psi; curtain air (CUR): 35 psi; ion source temperature (TEM): −550℃; and ion source voltage (IS): −4,500 V. The First‐level scan: declustering voltage (DP ): 100 V; focusing Voltage (CE): 10 V; the secondary scan: TOF MS‐Product Ion‐IDA mode was used to collect mass spectrum data, and CID energy was −20, −40 and −60 V. The CDS pump was used for mass axis correction to make it less than 2 ppm before sample injection.

#### 2.8.4 Structural analysis of compounds

According to the domestic and foreign literature reports on the phytochemical chemical constituents of *R. himalense*, and its same family and genera. At the same time, with the help of Scifinder and Reaxys databases, various chemical constituents in the fruit of *R. himalense* were collected. After using liquid mass spectrometry to collect data and extract the mass spectra of each chromatographic peak, according to the quasi‐molecular ion ([M + H]^+^, [M + H]^‐^) and the charged ion ([M+NH_4_]^+^, [M+Na]^+^, [M+Cl]^‐^) information judged and obtained the accurate relative molecular mass of the primary mass spectrum, and the molecular formula is fitted within the mass deviation range of 5 × 10^−6^ through the peakview 1.2 software, and compare with the literature database which makes a preliminary guess for each chromatographic peak. Secondary mass spectrometry with good signal‐to‐noise ratio was screened to obtain its information of chromatographic peaks, and corresponding fragment ions of compounds were obtained. Then the chemical composition was further predicted according to the fragmentation of ions and combined with literature data.

### 2.9 Assay of antioxidant activity in vitro

The antioxidant activities of the 30%, 60%, and 95% ethanol extracts of *R. himalense* fruits were determined microassay, using the T‐AOC assay (DPPH, ABTS, and FRAP) kits purchased from Suzhou Comin Biotechnology Co., Ltd. (Jiangsu, China). The scavenging capability for DPPH free radical and ABTS radical cation (ABTSˑ^+^) was evaluated by applying a microplate reader in absorbance at 515 and 734 nm, respectively (Abreu et al., 2019; Arts et al., 2004; Souza et al., 2020). The results of DPPH and ABTS were expressed as trolox equivalent antioxidant capacity (TEAC), which is µmol TEAC/g fresh weight and determined via a calibration curve, such as y = 0.7072x − 0.0081, *R* ^2^ = 0.9977 and y = 0.7021x − 0.0012, *R* ^2^ = 0.9985. The antioxidant activity was conducted using ferric reducing antioxidant power (FRAP) assay method, that is, under acidic conditions, the ability of antioxidants to reduce Fe^3+^‐TPTZ (2,4,6‐tripyridyl‐s‐triazine) to produce blue Fe^2+^‐TPTZ (Asghar et al., 2019; Oikeh et al., 2016; Razak et al., 2015). The absorbance was measured at 593 nm, and the FRAP results were represented as Fe (II) equivalent antioxidant capacity, which is µmol Fe (II)/g fresh weight, and determined via a standard curve (y = 1.2416x + 0.0134, *R* ^2^ = 0.9996). The analyses were carried out in triplicate for each concentration.

#### 2.9.1 Statistical analysis

The experimental data used statistical software SPSS 20.0 for variance analysis, Duncan's multiple range test is used to separate the means, and the results are reported as mean ± *SD*. A *p* value of .05 is considered a statistically significant difference. Origin 8.5 software was used to draw graphs.

## 3 RESULTS AND DISCUSSIONS

### 3.1 Proximate analysis and bioactive compounds contents

Proximate composition and phytochemical component of *R*. *himalense* fruit are presented in Table 1. The moisture content (81.35 ± 0.21 g/100 g FW). Protein is an essential nutrient in the human diet. From Table 1, we can see that protein content (1.81 ± 0.21 g/100 g FW)​ in *R*. *himalense* fruit. The crude fiber content (7.41 ± 0.40% FW) can be used as a good plant source of dietary fiber. Dietary fiber is an indigestible component in plant cell walls and plays an important role in human health, such as prevent or treat cardiovascular disease, hypertension, and diabetes (Ijarotimi et al., 2013).

**TABLE 1:** The content of proximate composition, bioactive compounds, minerals, and vitamins in *R. himalense* fresh fruit

Test items	Mean ± *SD*	Test items	Mean ± *SD*	Test items	Mean ± *SD*
Moisture (g/100g)	81.35 ± 0.21	Anthocyanin (mg/100g)	37.34 ± 0.75	Zn (mg/100g)	0.58 ± 0.03
Protein (g/100g)	1.81 ± 0.21	Procyanidine (%)	0.72 ± 0.10	Mn (mg/100g)	0.16 ± 0.03
Fat (g/100g)	2.38 ± 0.15	K (mg/100g)	218.44 ± 0.52	Vitamin C (mg/100g)	64.6 ± 1.21
Crude fiber (%)	7.41 ± 0.40	Ca (mg/100g)	56.46 ± 0.68	Vitamin B1 (mg/100g)	0.05 ± 0.02
Total flavonoid (%)	0.38 ± 0.07	Mg (mg/100g)	16.33 ± 0.64	Vitamin B2 (mg/100g)	0.05 ± 0.02
Polyphenolic (%)	0.49 ± 0.12	Cu (mg/100g)	2.67 ± 0.11	α‐Tocopherol (mg/100g)	0.25 ± 0.05
Polysaccharide(g/100g)	0.68 ± 0.15	Fe (mg/100g)	0.71 ± 0.03	γ‐Tocopherol (mg/100g)	0.09 ± 0.01

The phytochemical components in *R. himalense* fruit were as follows: phenolics (0.49 ± 0.12%), flavonoids (0.38 ± 0.07%), polysaccharide (0.68 ± 0.15%), anthocyanin (37.34 ± 0.75 mg/100 g), and procyanidine (0.72 ± 0.10%). According to previous literature reports, *Ribes nigrum* juice contains polyphenols (580.4 ± 0.86 mg GAE/100 g FW), flavonoids (84.6 ± 0.71 mg QE/100 g FW), and anthocyanin (116.1 ± 0.59 mg C3GE/100 g FW), while during ripening, total phenolic content of black currants (Rosenthal, Tenah, Titania) varies from 393 to 734 mg GAE/100g, anthocyanins from 196 to 461 mg C3GE/100g, and total phenolic value of red currants (Jonkheer van Tets, Junifer, Rovada) changes from 104 to 327 mg GAE/100g, for gooseberries (Achilles, Hinnonmaki gelb, Remarka) from 101 to 192 mg GAE/100g (Diaconeasa et al., 2015; Mikulic‐Petkovsek et al., 2015). Hence, the difference could be ascribed the extraction method used during juice preparation and also explained via cultivars, genetic differences, sampling dates, and environmental factors, such as growth conditions, season, and fruit maturity (Gavrilova et al., 2011; Mattila et al., 2016; Mikulic‐Petkovsek et al., 2012; Vagiri et al., 2013; Zheng et al., 2012). These compounds are found in abundance in *Ribes* berries and contain active molecules that have health benefits. Furthermore, phenolics, flavonoids, polysaccharide, anthocyanin, and procyanidine have been reported to possess antioxidant, anti‐inflammatory, anticancer, and antihyperglycemia properties (Braga et al., 2018; Ieri et al., 2015). These data have proved that *R. himalense* fruits contain both nutrient and bioactive substances, it will satisfy in which natural and functional.

### 3.2 Mineral compositions

Table 1 shown the mineral content of fruit from *R*. *himalense*. Minerals are classified into two groups: macroelements (K, Ca, and Mg) and microelements (Fe, Zn, Mn, and Cu). In this study, the most abundant macroelement was K (mean value of 218.44 mg/100 g FW), Ca (mean value of 56.46 mg/100 g FW), and Mg (mean value of 16.33 mg/100 g FW). The most abundant microelement was Cu (average content of 2.67 mg/100 g FW), Fe (average content of 0.71 mg/100 g FW), Zn (average content of 0.58 mg/100 g FW), and Mn (average content of 0.16 mg/100 g FW). Parrilla (*Ribes magellanicum* Poir., *Saxifragaceae*) fruits had higher K (234.00–238.00 mg/100 g FW), Ca (87.80–104.67 mg/100 g FW), and Fe (1.51–2.02 mg/100 g FW) values than *R*. *himalense* fruits from China, while Zn (0.10–0.11 mg/100 g FW) content lower than it (Damascos et al., 2008). The difference in mineral content in *Ribes* fruits may be affected by soils, climate, and other factors that influence the growth rate of plants, thereby affecting the utilization and loss ratio of mineral ions (Plessi et al., 2007; Sánchez‐Castillo et al., 1998).

### 3.3 Vitamin content

The vitamin content of *R. himalense* fresh fruit from wild is presented in Table 1. With an average of 64.6 ± 1.21 mg/100 g FW, vitamin C (ascorbic acid) was the most abundant vitamin in *R. himalense* samples. α‐Tocopherol (one of vitamin E is sum of tocopherols) was the second one with 0.25 ± 0.05 mg/100 g FW and γ‐tocopherol the third with 0.09 ± 0.01 mg/100 g FW. With the same average of 0.05 ± 0.02 mg/100 g FW, vitamin B1 and vitamin B2 were the vitamins with lowest amount found in the samples. The contents of ascorbic acid and vitamin E in various gooseberry and currant cultivars ranged from 6.2 to 14.04 g/kg and 0.43 to 12.85 mg/kg, respectively, and also known as an important source of vitamin C (Orsavová et al., 2019). Moreover, jostaberry (42.27 ± 6.63 mg/100 g FW), blueberry (12.60 ± 2.79 mg/100 g FW), and apple (3.91 ± 0.48 mg/100 g FW) display a lower ascorbic acid level than *Ribes himalense*, which has been reported in previous studies (Donno et al., 2015, 2018). Furthermore, content of vitamin C reached 64.6 mg/100g, in fruit market; vitamin C has been corresponding to more than 65% of antioxidant and antiviral activity in many fruits and their beverages (Mditshwa et al., 2017; Padayatty et al., 2003). Thus, the high content of vitamin C in this fruit makes it suitable for further development and application in commercial market.

### 3.4 Amino acid profiles

Amino acids (AA), the most important chemical elements in the world, are divided into essential amino acids and nonessential amino acids. The essential amino acids, defined as one that the body cannot make in sufficient amounts to maintain growth or nitrogen balance, must be absorbed from food and supply human body (Rose et al., 1948; Rose & Smith, 1949). AAs are important fundamental units of vital tissues, proteins and peptides (including enzymes and hormones), neurotransmitters, nourishment, and transporters. Thus, they are arousing great scientific interest for the researchers (Wahl & Holzgrabe, 2016).

Table 2 shown the amino acids profile of *Ribes himalense* fruit. The most abundant amino acid of *R. himalense* fruit sample was glutamic acid with an average content 0.35 ± 0.02 g/100g, followed by arginine (0.15 ± 0.02 g/100 g), aspartic acid (0.19 ± 0.01 g/100 g), leucine (0.12 ± 0.03 g/100 g), and valine (0.08 ± 0.01 g/100 g). Tryptophan with 0.08 ± 0.01 g/100 g and cysteine with 0.01 g/100 g were the amino acids with lower presence.

**TABLE 2:** The content of amino acids in *R. himalense* fresh fruit materials (g/100g)

Amino acids	Mean ± *SD*	Amino acids	Mean ± *SD*	Amino acids	Mean ± *SD*
Asp	0.19 ± 0.01	Pro	0.11 ± 0.03	His	0.16 ± 0.01
Glu	0.35 ± 0.02	Ala	0.09 ± 0.01	Lys	0.14 ± 0.02
Ser	0.12 ± 0.01	Val	0.08 ± 0.01	Tyr	0.08 ± 0.01
Gly	0.09 ± 0.01	Ile	0.11 ± 0.03	Met	ND
Arg	0.15 ± 0.02	Leu	0.12 ± 0.03	Cys	0.01 ± 0.00
Thr	0.08 ± 0.01	Phe	0.21 ± 0.03		

Abbreviation: ND, not detected.

This is the first study analyzing the nutritional profile of *R*. *himalense* samples from undeveloped wild area of Qinghai Plateau. Although we could observe differences among all samples, in general, the variance found between the nutritional compositions of the analyzed samples was not of great significance. Additionally, *Ribes* species berries are a good source of minerals (calcium, selenium), vitamins (VA, VC, VE), provitamins, and related compounds (carotene, lutein), which have an active role in dietary administration of various ailments, such as cardiovascular disease, cancer, osteoporosis, and inflammation (Laczkó‐Zöld et al., 2018). Therefore, as an undeveloped wild small berry, it is necessary to study the nutritional quality of *R*. *himalense*.

### 3.5 **Fr1 compounds in the fruit of *R***. ***himalense***

The UV spectrum and total ion chromatograms were obtained by qualitative analysis of *R. himalense* extract Fr1 with UPLC‐Triple‐TOF‐MS/MS is shown in Figure S1. According to the pyrolysis rules in the literature, combined with retention time data, using UV spectrum characteristics, and mass ion fragmentation, 61 chromatographic peaks were identified and their structures were derived, and their primary and secondary mass spectrometry were shown in Appendix (Figures S4–S64). These compounds included twenty‐five phenolic acids (2–10, 12, 14, 15, 20–22,24, 34, 36–38, 49, 54, 58–60), twenty flavonoids (16, 18, 25, 27, 42–53,55–57,61), five catechins (11, 13, 17, 23, 31), three anthocyanins (26, 28, 29), three organic acids (1, 19, 32), and five other ingredients (30, 35, 39–41), which were reported for the first time in Ribes himalense extract. The MS spectrometry results and fragmentation characteristics of the components of Fr1 are shown in Table 3.

**TABLE 3:** Retention times and characteristic ions of Fr 1 compounds of *R. himalense* fruit

Peak no.	Ion mode	Rt (min)	[M‐H]^‐^(m/z)	Proposed formula	Exact Mass	Measured mass	Δm (ppm)	MS/MS fragment(m/z)	Compound
1	‐	1.57	191.0207	C_6_H_8_O_7_	192.0270	192.0286	−0.0016	173.0141,129.0201	citric acid
2	‐	3.77	169.0157	C_7_H_6_O_5_	170.0215	170.0236	−0.0021	151.0054,125.0265	2,3,4‐trihydroxybenzoic acid
3	‐	3.89	315.0717	C_13_H_16_O_9_	316.0794	316.0796	−0.0002	153.0185,109.0312	protocatechuic acid 3'‐O‐β‐D‐glucopyranoside
4	‐	4.32	329.0872	C_14_H_18_O_9_	330.0951	330.0951	0.0000	167.0349,152.0114,123.0453	vanillic acid 1‐O‐β‐D‐glucopyranosyl ester
5	‐	4.63	153.0215	C_7_H_6_O_4_	154.0266	154.0294	−0.0028	109.0295,91.0192	3,4‐Dihydroxybenzoic acid
6	‐	4.79	357.0816	C_15_H_18_O_10_	358.0900	358.0895	0.0005	195.0506,179.0342,135.0455	2‐O‐trans‐caffeoylgluconic acid
7	‐	5.56	353.0872	C_16_H_18_O_9_	354.0951	354.0951	0.0000	191.0554,179.0342,135.0448	neochlorogenic acid
8	‐	5.96	503.1389	C_21_H_28_O_14_	504.1479	504.1468	0.0011	341.0894,179.0.350,135.0456	β‐D‐glucopyranosyl 4‐O‐β‐D‐glucopyranosylcaffeate
9	‐	6.00	329.0874	C_14_H_18_O_9_	330.0951	330.0953	−0.0002	209.0455,167.0347,152.0106	4‐O‐β‐D‐glucopyranosylvanillic acid
10	‐	6.39	341.0874	C_15_H_18_O_9_	342.0951	342.0953	−0.0002	179.0340,161.0240	4‐O‐β‐glucopyranosyl‐(E)‐caffeic acid
11	‐	6.87	577.1328	C_30_H_26_O_12_	578.1424	578.1407	0.0017	425.0874,407.0871,289.0712,125.0235	procyanidin B1
12	‐	7.20	341.0871	C_15_H_18_O_9_	342.0951	342.0950	0.0001	195.0502,163.0392	3,5,7‐trihydroxychromone 3‐O‐α‐L‐rhamnopyranoside
13	‐	7.48	577.1327	C_30_H_26_O_12_	578.1424	578.1406	0.0018	425.0864,407.0768,289.0706,125.0245	procyanidin B3
14	‐	7.58	357.0816	C_15_H_18_O_10_	358.0900	358.0895	0.0005	195.0506,179.0339,135.0447	6‐O‐trans‐caffeoylgluconic acid
15	‐	7.75	337.0921	C_16_H_18_O_8_	338.1002	338.1000	0.0002	191.0544,163.0394,119.0497	3‐O‐p‐coumaroylquinic acid
16	‐	8.00	463.0865	C_21_H_20_O_12_	464.0955	464.0944	0.0011	175.0235	eriodictyol‐7‐O‐β‐D‐glucuronopyranoside
17	‐	8.14	289.0722	C_15_H_14_O_6_	290.0790	290.0801	−0.0011	245.0824,203.0709,151.0392,123.0448	catechin
18	‐	8.26	465.1019	C_21_H_20_O_12_	466.1111	466.1098	0.0013	303.0505,285.0394	taxifolin 3‐O‐β‐D‐glucopyranoside
19	‐	8.40	443.1903	C_21_H_32_O_10_	444.1995	444.1982	0.0013	119.0348,189.1274,59.0184	dihydrophaseic acid 3'‐O‐β‐D‐glucopyranoside
20	‐	8.69	353.0864	C_16_H_18_O_9_	354.0951	354.0943	0.0008	191.0554	chlorogenic acid
21	‐	8.91	325.0922	C_15_H_18_O_8_	326.1002	326.1001	0.0001	163.0404,145.0308,119.0511	p‐coumaric acid glucosyl ester
22	‐	9.33	353.0870	C_16_H_18_O_9_	354.0951	354.0949	0.0002	191.0551,179.0343,173.0450,135.0449,93.0355	4‐O‐caffeoylquinic acid
23	‐	9.46	577.1327	C_30_H_26_O_12_	578.1424	578.1406	0.0018	451.1040,425.0874,407.0777,289.0711,125.0239	procyanidin B2
24	‐	9.52	179.0367	C_9_H_8_O_4_	180.0423	180.0446	−0.0023	135.0448,107.0498	caffeic acid
25	‐	9.66	743.1997	C_32_H_40_O_20_	744.2113	744.2076	0.0037	633.1725,607.1926	cyanidin 3‐O‐(2G‐xylosylrutinoside)‐water‐added derivatives
26	‐	9.95	447.0911	C_21_H_21_O_11_	449.1078	448.0990	1.0088	285.0392	cyanidin 3‐glucoside
27	‐	10.72	465.1012	C_21_H_22_O_12_	466.1111	466.1091	0.0020	285.0396	(2R,3R)‐2,3‐dihydroquercetin 7‐β‐D‐glucopyranoside
28	‐	10.88	593.1479	C_27_H_31_O_15_	595.1657	594.1558	1.0099	285.0402	cyanidin 3‐rutinoside
29	‐	10.88	725.1906	C_32_H_39_O_19_	727.2080	726.1985	1.0095	285.0381	cyanidin 3‐O‐(2(G))‐xylosylrutinoside
30	‐	11.33	401.1434	C_18_H_26_O_10_	402.1526	402.1513	0.0013	269.1024	benzyl β‐primeveroside
31	‐	11.67	289.0715	C_15_H_14_O_6_	290.0790	290.0794	−0.0004	245.0808,203.0700,151.0394,123.0451	epicatechin
32	‐	11.79	336.1079	C_16_H_19_NO_7_	337.1161	337.1158	0.0003	292.1181,200.0703	glucoindol A
33	‐	11.83	449.1068	C_21_H_22_O_11_	450.1162	450.1147	0.0015	287.0553	dihydrokaempferol‐3‐O‐β‐D‐glucopyranoside
34	‐	12.69	339.1081	C_16_H_20_O_8_	340.1158	340.1160	−0.0002	177.0554	4‐(3'‐glucopyranosyloxy‐4'‐hydroxyphenyl)‐3‐buten‐2‐one
35	‐	13.15	415.1596	C_19_H_28_O_10_	416.1682	416.1675	0.0007	269.1016	1′‐O‐benzyl‐α‐L‐rhamnopyranosyl‐(1″→6′)‐β‐D‐glucopyranoside
36	‐	13.55	163.0422	C_9_H_8_O_3_	164.0473	164.0501	−0.0028	119.0512	p‐Coumaric Acid
37	‐	13.75	167.0370	C_8_H_8_O_4_	168.0423	168.0449	−0.0026	152.0111,108.0226	3‐methoxy‐4‐hydroxybenzoic acid
38	‐	14.42	479.1895	C_24_H_32_O_10_	480.1995	480.1974	0.0021	317.1368,299.1282	albiflorin
39	‐	15.27	395.1900	C_17_H_32_O_10_	396.1995	396.1979	0.0016	249.1325	pentan‐2‐yl α‐L‐rhamnopyranosyl‐(1 → 6)‐β‐D‐glucopyranoside
40	‐	15.77	393.1749	C_17_H_30_O_10_	394.1839	394.1828	0.0011	251.0758	(Z)‐3‐hexenyl O‐β‐D‐xylopyranosyl‐(1''→6')‐β‐D‐glucopyranoside
41	‐	16.32	429.1749	C_20_H_30_O_10_	430.1839	430.1828	0.0011	265.0922	2‐phenylethyl O‐α‐L‐rhamnopyranosyl‐(1 → 6)‐β‐D‐glucopyranosidee
42	‐	16.54	741.0858	C_32_H_38_O_20_	742.1956	742.0937	0.1019	475.0893	isoorientin 7‐O‐glucoside 2''‐O‐arabinoside
43	‐	17.4	609.1434	C_27_H_30_O_16_	610.1534	610.1513	0.0021	301.0342	quercetin 3‐O‐α‐L‐rhamnopyranosyl(1 → 2)‐β‐D‐galactopyranoside
44	‐	17.51	609.1432	C_27_H_30_O_16_	610.1534	610.1511	0.0023	301.0345	quercetin‐(1β→7O)‐rutinoside
45	‐	17.53	463.0857	C_21_H_20_O_12_	464.0955	464.0936	0.0019	317.0295	myricitrin
46	‐	17.88	609.1432	C_27_H_30_O_16_	610.1534	610.1511	0.0023	301.0352	rutin
47	‐	17.95	463.0858	C_20_H_20_O_12_	464.0955	464.0937	0.0018	301.0342	hyperoside
48	‐	18.14	477.0655	C_21_H_18_O_13_	478.0747	478.0734	0.0013	301.0346	quercetin 3‐glucuronide
49	‐	18.37	521.1998	C_26_H_34_O_11_	522.2101	522.2077	0.0024	359.1496	7S,8R,8'R‐(‐)‐lariciresinol‐4‐O‐β‐D‐glucopyranoside
50	‐	18.48	463.0861	C_20_H_20_O_12_	464.0955	464.0940	0.0015	301.0348	isoquercetin
51	‐	19.43	433.0755	C_20_H_18_O_11_	434.0849	434.0834	0.0015	300.0265	avicularin
52	‐	19.98	433.0757	C_20_H_18_O_11_	434.0849	434.0836	0.0013	301.0354	quercetin 3‐O‐β‐D‐xylopyranoside
53	‐	20.08	505.0965	C_24_H_22_O_15_	506.1060	506.1044	0.0016	301.0349	6‐acetyl‐isoquercitrin
54	‐	20.34	333.061	C_16_H_14_O_8_	334.0689	334.0689	0.0000	301.0342,165.0192,137.0243,121.0291	2‐O‐(3,4‐dihydroxybenzoyl)‐2,4,6‐trihydroxyphenylmethylacetate
55	‐	20.68	433.0757	C_20_H_18_O_11_	434.0849	434.0836	0.0013	301.0355,271.0238,255.0289,243.0285	quercetin 3‐O‐L‐arabinopyranoside
56	‐	21.21	461.0700	C_21_H_18_O_12_	462.0798	462.0779	0.0019	285.0390	kaempferol 3‐O‐β‐D‐glucuronide
57	‐	22.62	601.0595	C_30_H_18_O_14_	602.0697	602.0674	0.0023	465.0472,301.0348	6,8''‐diquercetin
58	‐	25.40	301.0350	C_15_H_10_O_7_	302.0427	302.0429	−0.0002	165.0181	didyronic acid
59	‐	25.77	315.0502	C_16_H_12_O_7_	316.0583	316.0581	0.0002	283.0229	didyronic acid methyl ester
60	‐	26.96	683.1223	C_32_H_28_O_17_	684.1326	684.1302	0.0024	521.0732	puniceaside B
61	‐	27.81	301.0349	C_15_H_10_O_7_	302.0427	302.0428	−0.0002	273.0402,257.0427,178.9978,151.0033,121.0309,107.0145	quercetol

### 3.6 **Fr 2 and Fr 3 compounds in the fruit of *R***. ***himalense***

A sensitive and effective method based on UPLC‐Triple‐TOF‐MS/MS was established for the comprehensive analysis of chemical components in Fr 2 and Fr 3. The UV spectrum and total ion current map of Fr 2 and Fr 3 were obtained in 254 nm and negative ion mode (Figures S2 and S3), respectively. Through the application of chromatographic retention behaviors, [M‐H]^‐^ ions, mass fragmentation modes, and previous related literatures, 34 chemical constituents were identified from Fr 2 and Fr 3 extract, including eighteen phenolic acids (63–69, 72, 74–77, 79, 81, 82, 86, 95), seven flavonoids (71, 87–91, 93), nine other compounds (62, 70, 73, 78, 80, 83–85, 92, 94), and their MS and MS^2^ were shown in appendix (Figures S65–S98). Among them, 22 constituents were reported for the first time in *R. himalense* ethanol water extract (60:40, 95:5; v/v). Compounds were numbered by their elution order and summarized in Table 4.

**TABLE 4:** Retention times and characteristic ions of Fr 2 and Fr 3 compounds of *R. himalense* fruit

Peak no.	Ion mode	Rt (min)	[M‐H]^‐^ (m/z)	Proposed formula	Exact Mass	Measured mass	Δm (ppm)	MS/MS fragment(m/z)	Compound
62	‐	2.05	225.0416	C_10_H_10_O_6_	226.0477	226.0495	−0.0018	181.0496,163.0389	chorismic acid
63	‐	2.61	315.0724	C_13_H_16_O_9_	316.0794	316.0803	−0.0009	153.0182,109.0296	2‐hydroxylbenzoic acid‐5‐O‐β‐D‐glucopyranoside
64	‐	3.34	461.1290	C_19_H_26_O_13_	462.1373	462.1369	0.0004	153.0190,109.0305	gentisic acid‐5‐O‐α‐L‐rhamnopyranosyl‐(1 → 2)‐β‐D‐glucopyranoside
65	‐	3.53	315.0724	C_13_H_16_O_9_	316.0794	316.0803	−0.0009	153.0195,109.0309	5‐hydroxybenzoic acid‐2‐O‐glucopyranoside
66	‐	4.03	461.1286	C_19_H_26_O_13_	462.1373	462.1365	0.0008	153.0195,109.0305	gentisic acid 2‐O‐α‐L‐rhamnopyranosyl‐(1 → 2)‐β‐D‐glucopyranoside
67	‐	4.52	299.0771	C_13_H_16_O_8_	300.0845	300.085	−0.0005	137.0237	4‐(β‐D‐glucopyranosyloxy)hydroxybenzoic acid
68	‐	4.67	315.0722	C_13_H_16_O_9_	316.07943	316.0801	−0.0007	153.0189,135.0076, 109.0303	1‐O‐(2,5‐Dihydroxy‐benzoyl)‐β‐D‐glucopyranose
69	‐	5.71	137.0269	C_7_H_6_O_3_	138.0317	138.0348	−0.0031	93.0351	4‐hydroxybenzoic acid
70	‐	7.78	336.1088	C_16_H_19_NO_7_	337.1162	337.1167	−0.0005	292.1187	glucoindol A
71	‐	8.57	593.1506	C_27_H_30_O_15_	594.1585	594.1585	0.0000	473.1112,431.0999	Saponarin
72	‐	8.91	135.0482	C_8_H_8_O_2_	136.0524	136.0561	−0.0037	120.0210,108.0217,92.0278	3,4‐dihydroxystyrene
73	‐	9.91	429.1750	C_20_H_30_O_10_	430.1839	430.1829	0.0010	325.1114	2‐phenylethyl O‐α‐L‐rhamnopyranosyl‐(1 → 6)‐β‐D‐glucopyranoside
74	‐	10.39	489.1600	C_21_H_30_O_13_	490.1686	490.1679	0.0007	181.0506	2,6‐dihydroxy‐4‐methoxy‐acetophenone 2‐O‐β‐rutinoside
75	‐	10.91	521.201	C_26_H_34_O_11_	522.2101	522.2089	0.0012	359.1501	(4,9,4',9'‐tetrahydroxy‐3,3'‐dimethoxy‐8β,8'α,7'β‐cyclolignan)‐9'‐O‐β‐D‐glucopyranoside
76	‐	11.19	419.1696	C_22_H_28_O_8_	420.1784	420.1775	0.0009	257.1174	stilbostemin H 3'‐β‐D‐glucopyranoside
77	‐	12.92	457.17	C_21_H_30_O_11_	458.1788	458.1779	0.0009	293.0867	2‐methoxy‐5‐(E)‐propenyl‐phenol‐β‐vicianoside
78	‐	13.99	409.2068	C_18_H_34_O_10_	410.2152	410.2147	0.0005	277.1659,161.0446	isoheptanol 2(S)‐O‐β‐D‐xylopyranosyl‐(1 → 6)‐O‐β‐D‐glucopyranoside
79	‐	14.48	281.1187	C_18_H_18_O_3_	282.1256	282.1266	−0.0010	93.0362	ribesin A
80	‐	14.95	421.2091	C_19_H_34_O_10_	422.2152	422.217	−0.0018	289.1648	3‐O‐[α‐L‐arabinopyranosyl‐(1 → 6)‐β‐D‐glucopyranosyl]oct‐1‐ene‐3‐ol
81	‐	15.23	283.1343	C_18_H_20_O_3_	284.1412	284.1422	−0.0010	177.0914	larreatricin
82	‐	15.35	421.2069	C_19_H_34_O_10_	422.2152	422.2148	0.0004	289.1649	3‐O‐[α‐L‐arabinopyranosyl‐(1 → 6)‐β‐D‐glucopyranosyl]oct‐1‐ene‐3‐ol
83	‐	15.36	283.1343	C_18_H_20_O_3_	284.1412	284.1422	−0.0010	177.0912	3,3'‐didemethoxynectandrin B
84	‐	16.92	417.2481	C_21_H_38_O_8_	418.2567	418.256	0.0007	255.1944	(3S,6E,10R)‐11‐β‐D‐glucopyranosyloxy‐3,10‐dihydroxy‐3,7,11‐trimethyldodeca‐1,6‐diene
85	‐	17.02	447.222	C_21_H_36_O_10_	448.2308	448.2299	0.0009	315.1811	(R)‐linalool 6‐O‐α‐L‐arabinopyranosyl‐β‐D‐glucopyranoside
86	‐	6.02	137.027	C_7_H_6_O_3_	138.0317	138.0349	−0.0032	93.0347,65.0469	salicylic acid
87	‐	7.68	609.1442	C_27_H_30_O_16_	610.1534	610.1521	0.0013	447.0933,327.0516	7‐O‐β‐D‐glucopyranosyl‐6‐C‐β‐D‐glucopyranosylluteolin
88	‐	8.78	593.1501	C_27_H_30_O_15_	594.1585	594.158	0.0005	473.1116,431,1,005,341.0668,311.0559	Saponarin
89	‐	8.96	739.2078	C_33_H_40_O_19_	740.2164	740.2157	0.0007	431.1002,341.0668,311.0569	isovitexin 7‐rhamnosylglucoside
90	‐	9.19	563.1397	C_26_H_28_O_14_	564.1479	564.1476	0.0003	473.1115,443.0996,413.0884	schaftoside
91	‐	9.34	623.1608	C_28_H_32_O_16_	624.1690	624.1687	0.0003	608.1445,503.1223,461.1133,371.0782	isoscoparin 7‐O‐β‐D‐glucoside
92	‐	11.88	144.0481	C_9_H_7_NO	145.0528	145.056	−0.0032	115.0428	Indole‐3‐carboxaldehyde
93	‐	12.05	769.1982	C_37_H_38_O_18_	770.2058	770.2061	−0.0003	431.0999,341.0666,311.0558	isovitexin 2″‐O‐(6‴‐(E)‐feruloyl)glucopyranoside
94	‐	17.52	193.101	C_8_H_19_O_3_P	194.1072	194.1089	−0.0017	78.9596	n‐octylphosphonic acid
95	‐	20.72	191.0726	C_11_H_12_O_3_	192.0786	192.0805	−0.0019	163.0391,145.0280	epi‐isoshinanolone

### 3.7 Phenolic acids and derivative

Compound **24** was identified as caffeic acid based on [M‐H]^‐^ ion at m/z 179.0367, and product ions at m/z 135 [M‐44‐H] and 107 [M‐44‐28‐H], which is consistent with the reported data of this compound (Santos et al., 2012). Compounds **7**, **20** and **22** showed similar [M‐H]^‐^ ions at m/z 353.0872, 353.0864, and 353.0870, the same molecular formula C_16_H_18_O_9_, the same product ions at m/z 191,179,135, but their retention times are different, which were putatively identified as neochlorogenic acid, chlorogenic acid, and 4‐*O*‐caffeoylquinic acid, respectively (Table 3). Compounds **81** and **83** showed same [M‐H]^‐^ ions at m/z 283.1343, the same chemical formula C_18_H_20_O_3_, the same fragmentation pattern at m/z 77 [M‐106‐H], due to phenyl migration, while their retention times are different, which were tentatively identified as larreatricin and 3,3'‐didemethoxynectandrin B, respectively (Table 4).

Compound **9** was identified as 4‐*O*‐*β*‐*D*‐glucopyranosyl vanillic acid, due to its [M‐H]^‐^ ion at m/z 329.0874 yielding its characteristic fragmentation ions at m/z 209 [M‐120‐H], 167 [M‐162‐H], and 152 [M‐162‐15‐H]. Compounds **10** and **12** were identified as 4‐*O*‐*β*‐glucopyranosyl‐(*E*)‐caffeic acid and 3,5,7‐trihydroxychromone 3‐*O*‐*α*‐*L*‐rhamnopyranoside, they have the same molecular formula C_15_H_18_O_9_, but their [M‐H]^‐^ ions are slightly different, and retention times and characteristic product ion are obviously different. Similarly, compounds **64** and **66** show the same molecular formula C_19_H_26_O_13_,with a difference of 0.0004 in the value of [M‐H]^‐^ ions and produce the same characteristic fragment ions at m/z 153 [M‐308‐H] and 109 [M‐308‐44‐H] (loss of rhamnoglycosyl and carboxyl groups), which are preliminarily inferred as gentisic acid‐5‐*O*‐*α*‐*L*‐rhamnopyranosyl‐(1 → 2)‐*β*‐*D*‐glucopyranoside, gentisic acid 2‐O‐*α*‐L‐rhamnopyranosyl‐(1 → 2)‐*β*‐D‐glucopyranoside, due to their inconsistent retention time. On the other hand, compounds **63** and **65** were identified as hydroxylbenzoic acid glucopyranoside isomers, due to their [M‐H]^‐^ at m/z 315.0724, which fragmented in m/z 153 [M‐162‐H] and 109 [M‐162‐44‐H], owing to the loss of glucosyl and carboxyl. Peaks **6**, **8**, and **14** [M‐H]^‐^ ions are at m/z 357.0816, 503.1389, and 357.0816, respectively, which are broken at m/z 179 because of the loss of hexose (162 Da) residues. Therefore, based on their retention time and product ions, these compounds were preliminarily identified as caffeoylhexose isomers compared with the Scifinder and Reaxys mass spectrometry library. Clifford et al. (2003) and Clifford et al. (2005) suggested that the isomers of caffeylquinic acid could be distinguished according to their cleavage patterns and the intensity of the fragmented ion in the mass spectrum. The MS/MS fragment of coumaroylquinic acid produced [M‐H]^‐^ ion at m/z 353, which was due to the loss of deoxyhexose (146 Da), and m/z was at 337 due to hexose (162 Da) loss, and the fragments with m/z of 191, 179, and 163, corresponding to quinic, caffeic (Peak **24**) and *p*‐coumaric acids (Peak **36**), respectively (Guarnerio et al., 2011). Peak **15** was identified as 3‐*O*‐*p*‐coumaroylquinic acid based on [M‐H]^‐^ ion at m/z 337.0921, and on the typical fragmentation ions in negative ion mode at m/z 191,163, and 119, and according to retention time at 7.75 min. The health benefits proposed by previous studies are usually related to phenols, which are the main biologically active compounds of *Ribes* fruits (Zdunić et al., 2016).

### 3.8 Flavonoids

Based on Scifinder and Reaxy databases and the literatures, the major aglycones were quercetin, cyanidin, taxifolin, isorhamnetin, isoorientin, myricitrin, quercetol, eriodictyol, and kaempferol in *R. himalense* fruit. According to the MS/MS fragmentation ions informatics and retention time, quercetin found a typical fragment at m/z 301; with characteristic ions at m/z 317, it could be inferred that the aglycone was isorhamnetin; and the specific fragment in 287 Da was caused by kaempferol. On the other hand, based on the cleavage mode of sugar residues, the number and types of glycosyl groups could be speculated.

Compounds **16**, **18**, **25**, **27**, **33**, **42–48**, **50–53**, and **55–57** were characterized as *O*‐glycoside and *O*‐glucuronide of flavones which all showed ions at m/z [aglycone‐H]^‐^ in the MS^n^ spectra in negative ion mode with the loss of a neutral rhamnosyl unit, arabinosyl unit, galactosyl unit, and glucosyl unit. Compounds **47** and **50** were identified as quercetin 3‐*O*‐galactoside (hyperoside) and quercetin 3‐*O*‐glucoside (isoquercetin), based on their [M‐H]^‐^ at m/z 463.0858, and the corresponding loss of 162 mass units, which indicates the existence of a hexose unit. In addition, the observed cleavage pathways, UV spectra, and retention times were matched with literature data (Galvis Sánchez et al., 2003). Peak **46** [M‐H]^‐^ ion at m/z 609.1432 fragmented in m/z 301, due to the loss of rhamnose (146 Da) and glucose (162 Da) residues, which was identified as quercetin 3‐*O*‐rutinoside (rutin). Compound **53** showed an [M − H]^−^ ion at m/z 505.0965, which yielding characteristic product ions at m/z 301 [M‐162‐42‐H], owing to the loss of hexoside residue (162 Da) and acetyl group (42 Da). This fragmentation mode is consistent with isoquercetin acylated hexoside (Guimarães et al., 2013). Compounds **16**, **48**, and **56** were referred to be eriodictyol‐7‐*O*‐*β*‐*D*‐glucuronopyranoside, quercetin 3‐*O*‐*β*‐*D*‐glucuronide, and kaempferol 3‐*O*‐*β*‐*D*‐glucuronide according to the MS^2^ data and literature, respectively.

Compounds **71**, **87–91**, and **93** were identified as *C*‐glycoside of flavones which shared typical fragment ions at m/z [M‐120‐H]^‐^, [M‐90‐H]^‐^, [M‐162‐90‐H]^‐^, and [M‐162‐120‐H]^‐^ in the MS/MS spectra in negative ion mode. These fragment ions were consistent with the literature (Wang et al., 2014). Thus, compounds **71**, **87–91**, and **93** were assigned as apigenin 6‐*C*‐glucosyl‐7‐*O*‐glucoside, 7‐*O*‐glucopyranosyl‐6‐*C*‐glucopyranosylluteolin, isovitexin 7‐*O*‐rhamnosylglucoside, schaftoside, isoscoparin 7‐*O*‐glucoside, and isovitexin 2″‐*O*‐(6‴‐(*E*)‐feruloyl)‐glucopyranoside, respectively.

### 3.9 Flavan‐3‐ols

Compound **17** was assigned as (+)‐catechin based on its [M‐H]^‐^ ion at m/z 289.0722, retention time at 8.14 min, and diagnostic ions, which in accordance with the reference compound. Additionally, compound **31** with m/z at 289.0715 and retention time at 11.67 min was identified as (‐)‐epicatechin, which yielded typical product ions at m/z 245, 203, 151, and 123. The retention time of (‐)‐epicatechin stereoisomer is always higher than that of (+)‐catechin on C18 inverse‐phase column, which means that catechin is eluted earlier than epicatechin (Mena et al., 2012). Compounds **11**, **13**, and **23** were tentatively identified as B‐type procyanidin dimers, due to their [M‐H]^‐^ ions at m/z 577.1328, and characteristic fragmentation ions at m/z 407 and 289, in agreement with a previous study (Kolniak‐Ostek & Oszmiański, 2015; Liu et al., 2016; Rahman et al., 2018).

#### 3.9.1 Anthocyanins

UPLC‐Triple‐TOF‐MS/MS analysis revealed the presence of three anthocyanins in the studied *R*. *himalense* fruit extract, using a negative ionization mode. Anthocyanins were identified based on complementary information of chromatographic behavior and mass fragments, as well as UV spectra and retention time. Compound **26** was characterized as cyanidin 3‐*O*‐glucoside with [M‐H]^‐^ ion at m/z 447.0911, and its MS^2^ fragment ion at m/z 285 [M‐162‐H], owing to the loss of hexose (162 Da) and corresponding to cyanidin residue (Galvis Sánchez et al., 2003). Then, compound **28** was identified as cyanidin 3‐*O*‐rutinoside based on [M‐H]^‐^ ion at m/z 593.1479, and its MS^2^ fragment ion at m/z 285 [M‐308‐H], owing to the loss of hexose (162 Da) and deoxyhexose (146 Da) (Diaconeasa et al., 2019). Finally, compound **29** was identified as cyanidin 3‐*O*‐(2(G))‐xylosylrutinoside. Therefore, it can be seen that the anthocyanin of *R. himalense* fruit is composed of an aglycon canditin and three glycosides (including glucose, rutin, and xylose), that is, the main anthocyanins identified were glycosylated cyanidin derivatives. Furthermore, anthocyanins exhibit various health benefits such as antioxidation, antiradiation, antihyperlipidemic, and antigout arthritis (Zhang et al., 2019).

#### 3.9.2 Characterization of organic acids

Compound **1** was identified as citric acid based on the precursor ion at m/z 191.0207, which resulting product ions at 173 [M‐18‐H] and 129 [M‐44‐18‐H] in negative ion mode, due to typical fragment ions produced by the neutral loss of H_2_O and CO_2_, which indicated the presence of carboxyl and hydroxyl groups in the molecular structures. Similarly, compounds **19**, **32**, and **62** were putatively characterized as organic acids, which match diagnostic ions in MS^2^ with those reported in literature and Scifinder database.

#### 3.9.3 Characterization of other compounds

Fourteen other compounds were detected, including ten diglycosides, two monoglycosides, indole‐3‐carboxaldehyde, and *n*‐octylphosphonic acid. Peak **35** shows that the [M‐H]^‐^ ion is m/z 415.1596 in the primary mass spectrum, and the mass spectrometry software calculated the exact molecular formula as C_19_H_28_O_10_. Both fragment ions at m/z 269.1016 and m/z 161.0452 can be seen in the MS^2^ spectrum, corresponding to [M‐H‐Rha]^‐^ and [Glu‐H‐H_2_O]^‐^. Therefore, peak35 is presumed to be phenethyl rutin (1′‐*O*‐benzyl‐α‐L‐rhamnopyranosyl‐(1″→6′)‐*β*‐*D*‐glucopyranoside) (Zeng et al., 2017). In the same way, the other chromatographic peaks were conjectured.

#### 3.9.4 Antioxidant activity of *R. himalense* fruit extracts

**FIGURE 1:**
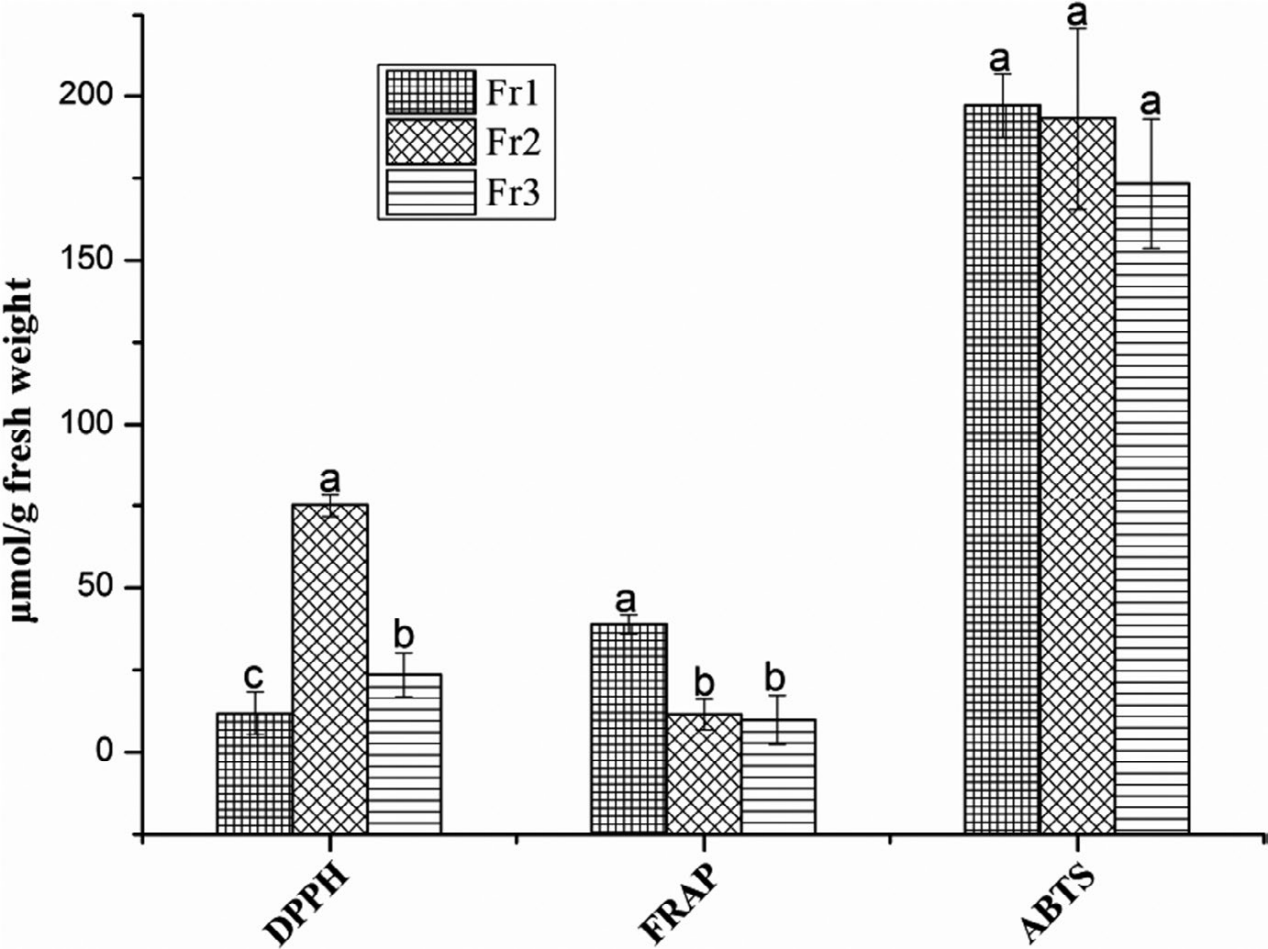
DPPH radical scavenging capability, ABTS radical scavenging ability, and ferric reducing antioxidant power (FRAP) of the three different ethanol extract samples from *R. himalense* fruits

In vitro antioxidant models, the DPPH and FRAP tests were commonly used to assess antioxidants in lipophilic and hydrophilic systems, respectively, yet the ABTS method used in both (Lee et al., 2011). The results of DPPH, ABTS, and FRAP assays are presented in Figure 1. All the three different ethanol extracts showed obvious scavenging abilities on the free radicals tests, and the scavenging activities varied remarkably in the different ethanol samples. The Fr2 (ethanol 60% extract) had the highest DPPH free radical scavenging ability at 75.07 ± 3.54 µmol TEAC/g FW, while the Fr3 (ethanol 95% extract) and Fr1 (ethanol 30% extract) at lower values of 23.50 ± 6.67 and 11.81 ± 6.39 µmol TEAC/g FW, respectively. Similarly, the scavenging abilities of ABTS free radical reduced in the following order: Fr1 (197.14 ± 9.90 µmol TEAC/g FW)> Fr2 (193.15 ± 27.65 µmol TEAC/g FW)> Fr3 (173.31 ± 19.59 µmol TEAC/g FW). The FRAP value of the Fr1 was 38.92 ± 2.82 μmol Fe (II)/g FW, which was notably higher than that of the Fr2 and Fr3 (*p* < 0.05). This demonstrated that the FRAP value declined by 70.50% and 74.78% in the Fr2 and Fr3, respectively.

The results in vitro showed that the *R. himalense* fruits could be effective sources of antioxidants. In addition, previous investigators had reported that the antioxidant capacity cannot be correlated just with the total polyphenol content of extracts, which is owing to the complex activity of various compounds, such as flavonoids and anthocyanins (Chai et al., 2020; Laczkó‐Zöld et al., 2018; Lin et al., 2016). Moreover, previous findings on *Ribes* species fruits, including *Ribes nigrum* L. (black currant), *Ribes rubrum* L. (red currant), currant cultivars, *Ribes uva‐crispa* L. (gooseberry), and *R. biebersteinii* Berl., indicated that these berries are the richest in polyphenols, such as anthocyanins, flavonoids, and phenolic acids, which play a vital role in the prevention and control of various illnesses through equilibriuming the oxidative and antioxidation factors in the human body (Carole et al., 2019; Delazar et al., 2010; Hurst et al., 2020; Laczkó‐Zöld et al., 2018). Therefore, phenolic compounds in the *R. himalense* fruits could act as a main contributor to their antioxidant activity, which was consistent with the result of total phenolic content measurements.

## 4 CONCLUSIONS

In summary, the structure of the detected compounds was identified by the following methods: (a) unambiguously recognized by comparing with reference standard compounds, such as analysis of nutrients content (including minerals, amino acids, vitamins); (b) characterized by cleavage pathways and typical fragment ions according to the relevant references; (c) preliminarily identified by searching Scifinder and Reaxy databases, like phenolic acids, flavonoids, proanthocyanidins, anthocyanins, and organic acids.

Berry is a fruit loved by consumers and is recognized as a food with multiple health benefits. In this work, we have carried out nutritional properties, chemical characterization, and antioxidant activities of the small berries of *R. himalense* picked from the plateau to clarify whether they is still a good source of this health promotion. According to the comparison with the literature data of the same genus fresh berry black currant, the nutritional profile showed that the wild fruit *R*. *himalense* has excellent nutritional value, with the high content of amino acids, vitamin C, and minerals (such as potassium, magnesium, and calcium), as well as contains a lot of various health‐enhancing substances, such as phenolic compounds. On the other hand, we used UPLC‐Triple‐TOF‐MS/MS to establish a reliable and effective analytical method for the separation and identification of various phytochemical components in the *R*. *himalense* fruit. Their chemical composition showed that 95 compounds, including 42 phenolic acids, 27 flavonoids, 5 proanthocyanidins, 4 organic acids, 3 anthocyanins, and 14 other substances, were initially identified, and their characteristic behaviors were described. Furthermore, the antioxidant activities of *R. himalense* ethanol extracts were determined by various methods (DPPH, ABTS, and FRAP), and obtained results in vitro showed that *R. himalense* berries are an excellent source of bioactive compounds with prospects of various health‐promoting functions, particularly phytochemicals performing considerable antioxidant capacity and conducing to the prophylaxis of some diseases caused by oxidative stress. As far as we know, this was the first report of systematic analysis of the chemical constituents and nutrients of *R*. *himalense* fruit. Based on the results obtained, because of its nutrition and chemical composition, the *R*. *himalense* proved to be a good choice for enriching the daily diet and could also be regarded as a rich natural source of nutrients with high antioxidant potential. Therefore, it should be widely used in modern nutritious food, cosmetics, and pharmaceutical industries to study functional products with potential health benefits.

## CONFLICTS OF INTEREST

The authors have declared no conflicts of interest for this article.

## AUTHOR CONTRIBUTION

Qing Sun: Conceptualization (lead); Data curation (lead); Investigation (equal); Software (equal); Supervision (lead); Writing‐original draft (lead). Na Wang: Data curation (supporting); Investigation (equal); Software (equal); Supervision (equal). Wenhua Xu: Investigation (supporting); Project administration (lead); Supervision (supporting). Huakun Zhou: Investigation (supporting); Project administration (equal); Resources (equal); Validation (equal).

## ETHICAL STATEMENT

This study does not involve any human or animal testing.

## Supporting information

Supplementary Material
