# Supplementary material for: Ribes himalense as potential source of natural bioactive compounds: Nutritional, phytochemical, and antioxidant properties
Source: Food Sci Nutr. 2021 May 3;9(6):2968–84. doi: 10.1002/fsn3.2256 (PMC8194758; doi:10.1002/fsn3.2256)
Supplement: Supplementary file 1 — Supplementary Material [file FSN3-9-2968-s001.doc]

**Figure S1 Fr1 UV (254nm) chromatogram and total ion current (negative) of 30% ethanol extract**

**Figure S2** **Fr2 UV (254nm) chromatogram and total ion current (negative) of 60% ethanol extract**

**Figure S3** **Fr3 UV (254nm) chromatogram and total ion current (negative) of 95% ethanol extract**

**Figure S4 The primary and secondary mass spectrum of citric acid**

**Figure S5 The primary and secondary mass spectrum of 2,3,4-trihydroxybenzoic acid**

**Figure S6 The primary and secondary mass spectrum of** **protocatechuic acid 3'-O-β-D-glucopyranoside**

**Figure S7 The primary and secondary mass spectrum of** **vanillic acid 1-O-β-D-glucopyranosyl ester**

**Figure S8 The primary and secondary mass spectrum of 3,4-Dihydroxybenzoic acid**

**Figure S9 The primary and secondary mass spectrum of 2-O-trans-caffeoylgluconic acid**

**Figure S10 The primary and secondary mass spectrum of neochlorogenic acid**

**Figure S11 The primary and secondary mass spectrum of β-D-glucopyranosyl 4-O-β-D-glucopyranosylcaffeate**

**Figure S12 The primary and secondary mass spectrum of 4-O-β-D-glucopyranosylvanillic acid**

**Figure S13 The primary and secondary mass spectrum of 4-O-β-glucopyranosyl-(E)-caffeic acid**

**Figure S14 The primary and secondary mass spectrum of procyanidin B1**

**Figure S15 The primary and secondary mass spectrum of 3,5,7-trihydroxychromone 3-O-α-L-rhamnopyranoside**

**Figure S16 The primary and secondary mass spectrum of procyanidin B3**

**Figure S17 The primary and secondary mass spectrum of 6-O-trans-caffeoylgluconic acid**

**Figure S18 The primary and secondary mass spectrum of 3-O-p-coumaroylquinic acid**

**Figure S19 The primary and secondary mass spectrum of eriodictyol-7-O-β-D-glucuronopyranoside**

**Figure S20 The primary and secondary mass spectrum of catechin**

**Figure S21 The primary and secondary mass spectrum of taxifolin 3-O-β-D-glucopyranoside**

**Figure S22 The primary and secondary mass spectrum of dihydrophaseic acid 3'-O-β-D-glucopyranoside**

**Figure S23 The primary and secondary mass spectrum of chlorogenic acid**

**Figure S24 The primary and secondary mass spectrum of p-coumaric acid glucosyl ester**

**Figure S25 The primary and secondary mass spectrum of 4-O-caffeoylquinic acid**

**Figure S26 The primary and secondary mass spectrum of procyanidin B2**

**Figure S27 The primary and secondary mass spectrum of caffeic acid**

**Figure S28 The primary and secondary mass spectrum of cyanidin 3-O-(2G-xylosylrutinoside)-water-added derivative**

**Figure S29 The primary and secondary mass spectrum of cyanidin-3-glucoside**

**Figure S30 The primary and secondary mass spectrum of (2R,3R)-2,3-dihydroquercetin 7-β-D-glucopyranoside**

**Figure S31 The primary and secondary mass spectrum of cyanidin-3-rutinoside**

**Figure S32 The primary and secondary mass spectrum of cyanidin 3-O-(2(G))-xylosylrutinoside**

**Figure S33 The primary and secondary mass spectrum of benzyl β-primeveroside**

**Figure S34 The primary and secondary mass spectrum of epicatechin**

**Figure S35 The primary and secondary mass spectrum of glucoindol A**

**Figure S36 The primary and secondary mass spectrum of dihydrokaempferol-3-O-β-D-glucopyranoside**

**Figure S37 The primary and secondary mass spectrum of 4-(3'-glucopyranosyloxy-4'-hydroxyphenyl)-3-buten-2-one**

**Figure 38 The primary and secondary mass spectrum of 1′-O-benzyl-α-L-rhamnopyranosyl-(1″→6′)-β-D-glucopyranoside**

**Figure S39 The primary and secondary mass spectrum of p-Coumaric Acid**

**Figure S40 The primary and secondary mass spectrum of 3-methoxy-4-hydroxybenzoic acid**

**Figure S41 The primary and secondary mass spectrum of albiflorin**

**Figure S42 The primary and secondary mass spectrum of pentan-2-yl α-L-rhamnopyranosyl-(1→6)-β-D-glucopyranoside**

**Figure S43 The primary and secondary mass spectrum of (Z)-3-hexenyl O-β-D-xylopyranosyl-(1''→6')-β-D-glucopyranoside**

**Figure S44 The primary and secondary mass spectrum of 2-phenylethyl O-α-L-rhamnopyranosyl-(1→6)-β-D-glucopyranoside**

**Figure S45 The primary and secondary mass spectrum of isoorientin 7-O-glucoside 2''-O-arabinoside**

**Figure S46 The primary and secondary mass spectrum of quercetin 3-O-α-L-rhamnopyranosyl(1→2)-β-D-galactopyranoside**

**Figure S47 The primary and secondary mass spectrum of quercetin-(1β→7O)-rutinoside**

**Figure S48 The primary and secondary mass spectrum of myricitrin**

**Figure S49 The primary and secondary mass spectrum of rutin**

**Figure S50 The primary and secondary mass spectrum of hyperoside**

**Figure S51 The primary and secondary mass spectrum of quercetin 3-glucuronide**

**Figure S52 The primary and secondary mass spectrum of 7S,8R,8'R-(-)-lariciresinol-4-O-β-D-glucopyranoside**

**Figure S53 The primary and secondary mass spectrum of isoquercetin**

**Figure S54 The primary and secondary mass spectrum of avicularin**

**Figure S55 The primary and secondary mass spectrum of quercetin-3-O-β-D-xylopyranoside**

**Figure S56 The primary and secondary mass spectrum of 6-acetyl-isoquercitrin**

**Figure S57 The primary and secondary mass spectrum of 2-O-(3,4-dihydroxybenzoyl)-2,4,6-trihydroxyphenylmethylacetate**

**Figure S58 The primary and secondary mass spectrum of quercetin 3-O-L-arabinopyranoside**

**Figure S59 The primary and secondary mass spectrum of kaempferol 3-O-β-D-glucuronide**

**Figure S60 The primary and secondary mass spectrum of 6, 8''-diquercetin**

**Figure S61 The primary and secondary mass spectrum of didyronic acid**

**Figure S62 The primary and secondary mass spectrum of didyronic acid methyl ester**

**Figure S63 The primary and secondary mass spectrum of puniceaside B**

**Figure S64 The primary and secondary mass spectrum of quercetol**

**Figure S65 The primary and secondary mass spectrum of chorismic acid**

**Figure S66 The primary and secondary mass spectrum of 2-hydroxylbenzoic acid-5-O-β-D-glucopyranoside**

**Figure S67 The primary and secondary mass spectrum of gentisic acid 5-O-α-L-rhamnopyranosyl-(1→2)-β-D-glucopyranoside**

**Figure S68 The primary and secondary mass spectrum of 5-hydroxybenzoic acid-2-O-glucopyranoside**

**Figure S69 The primary and secondary mass spectrum of gentisic acid- 2-O-α-L-rhamnopyranosyl-(1→2)-β-D-glucopyranoside**

**Figure S70 The primary and secondary mass spectrum of 4-(β-D-glucopyranosyloxy)hydroxybenzoic acid**

**Figure S71 The primary and secondary mass spectrum of 1-O-(2,5-Dihydroxy-benzoyl)-β-D-glucopyranose**

**Figure S72 The primary and secondary mass spectrum of 4-hydroxy-benzoic acid**

**Figure S73 The primary and secondary mass spectrum of glucoindol A**

**Figure S74 The primary and secondary mass spectrum of Saponarin**

**Figure S75 The primary and secondary mass spectrum of 3,4-dihydroxystyrene**

**Figure S76 The primary and secondary mass spectrum of 2-phenylethyl O-α-L-rhamnopyranosyl-(1→6)-β-D-glucopyranoside**

**Figure S77 The primary and secondary mass spectrum of 2,6-dihydroxy-4-methoxy-acetophenone 2-O-β-rutinoside**

**Figure S78 The primary and secondary mass spectrum of (4,9,4',9'-tetrahydroxy-3,3'-dimethoxy-8β,8'α,7'β-cyclolignan)-9'-O-β-D-glucopyranoside**

**Figure S79 The primary and secondary mass spectrum of stilbostemin H 3'-β-D-glucopyranoside**

**Figure S80 The primary and secondary mass spectrum of 2-methoxy-5-(E)-propenyl-phenol-β-vicianoside**

**Figure S81 The primary and secondary mass spectrum of isoheptanol 2(S)-O-β-D-xylopyranosyl-(1→6)-O-β-D-glucopyranoside**

**Figure S82 The primary and secondary mass spectrum of ribesin A**

**Figure S83 The primary and secondary mass spectrum of 3-O-[α-L-arabinopyranosyl-(1→6)-β-D-glucopyranosyl]oct-1-ene-3-ol**

**Figure S84 The primary and secondary mass spectrum of larreatricin**

**Figure S85 The primary and secondary mass spectrum of 3-O-[α-L-arabinopyranosyl-(1→6)-β-D-glucopyranosyl]oct-1-ene-3-ol isomer**

**Figure S86 The primary and secondary mass spectrum of 3,3'-didemethoxynectandrin B**

**Figure S87 The primary and secondary mass spectrum of (3S,6E,10R)-11-β-D-glucopyranosyloxy-3,10-dihydroxy-3,7,11-trimethyldodeca-1,6-diene**

**Figure S88 The primary and secondary mass spectrum of (R)-linalool 6-O-α-L-arabinopyranosyl-β-D-glucopyranoside**

**Figure S89 The primary and secondary mass spectrum of salicylic acid**

**Figure S90 The primary and secondary mass spectrum of 7-O-β-D-glucopyranosyl-6-C-β-D-glucopyranosylluteolin**

**Figure S91 The primary and secondary mass spectrum of Saponarin**

**Figure S92 The primary and secondary mass spectrum of isovitexin 7-rhamnosylglucoside**

**Figure S93 The primary and secondary mass spectrum of schaftoside**

**Figure S94 The primary and secondary mass spectrum of isoscoparin-7-O-β-D-glucoside**

**Figure S95 The primary and secondary mass spectrum of indole-3-carboxaldehyde**

**Figure S96 The primary and secondary mass spectrum of isovitexin 2″-O-(6‴-(E)-feruloyl)glucopyranoside**

**Figure S97 The primary and secondary mass spectrum of n-octylphosphonic acid**

**Figure S98 The primary and secondary mass spectrum of epi-isoshinanolone**
